# Shame, Stigma and HIV: Considering Affective Climates and the Phenomenology of Shame Anxiety

**DOI:** 10.34041/ln.v27.741

**Published:** 2021-11-04

**Authors:** Luna Dolezal

**Keywords:** Shame anxiety, stigma, HIV, affective climates, phenomenology

## Abstract

The affective climate often associated with HIV prevention and care practices is often dominated by negative emotions such as shame, fear and suspicion which arise because of HIV’s historical stigma. This article explores the experiential consequences of this affective climate and the continued stigma associated with HIV, through a focus on the experience of shame anxiety which can be understood as the chronic anticipation of shame or shameful exposure. Exploring first-person narratives of gay men living with HIV, the article gives an account of how shame anxiety is central to understanding how stigma causes harm, especially in experiences of chronic illnesses such as HIV. Using a philosophical framework, through phenomenology, it will be demonstrated how shame anxiety manifests in bodily lived experience through the structure of the “horizon”. The article will finish with reflections on how shame anxiety can act as a barrier to the effective delivery of health services for those with stigmatised chronic illnesses and, furthermore, why the experience of shame anxiety might be useful to consider when delivering health services.

## Introduction

**WHILE IT IS** widely recognised that HIV is a stigmatised condition and “reducing stigma” and creating “health-enabling environments” are important policy goals in relation to HIV prevention and treatment (Race 2018, 117), these goals remain abstract aims if not grounded in the bodily and affective lived experiences of individuals who face the social and personal difficulties inherent to HIV disclosure along with on-going health interventions and treatments. In his recent book *The Gay Science*, queer theorist Kane Race discusses a common “affective climate” often associated with HIV prevention and care practices as a result of HIV’s inherent stigma: it is “a shared context of fear, shame, secrecy, suspicion, rejection and avoidance…which materially and historically accumulates to make HIV prevention and care practices more (or less in this instance) possible” (Race 2018, 117). Race’s insights into the centrality of affect for the “contours of experience” and how affective experience in turn gives rise to “different practical possibilities (and indeed impossibilities)” in relation to sexual and social interactions, along with health-related care (Race 2018, 52), provide an inroad for investigating how HIV-related stigma manifests affectively in lived experience and its practical consequences.

While HIV stigma has been theorised and investigated extensively, there remains a curious lack of phenomenological clarity in the literature regarding how stigma manifests, or causes harm, in lived experience.^1^ As should be apparent by the definition of the term, stigma – broadly understood as a mark of disgrace that can discredit an individual and their social standing (Goffman 1990; Tyler 2020; Scambler 2020) – is not something that is experienced *directly* (as we might experience the pain or discomfort associated with illness). Instead, stigma is experienced *indirectly* through association. Interpersonal and social experiences such as labelling, social exclusion, vilification, discrimination, prejudice and judgement come to be read to be the result of the stigma associated with one’s illness. As a result, while the concept of health-related stigma has been thoroughly theorised and conceptualised, “the mechanism by which stigma may cause distress remains largely unknown,” as Bennett et al. have noted recently in their discussion of HIV (Bennett et al. 2016, 87). In short, stigma is a socio-political category which cannot be experienced *in itself*, and while “stigma experiences” (Stangl et al. 2019, 2), such as labelling, social exclusion etc., are clearly negative and potentially harmful, the bodily and affective first-person lived experience of stigma-related phenomena is often missing in discussions about stigma and stigma experiences.

Naturally, the emphasis on the social manifestations of stigma is important, such that the individual does not carry the burden or blame for the negative attributes associated with their stigmatised condition, and so that stigma is recognised as a structural and political (rather than individual) problem. However, if one seeks to understand the mechanisms through which stigma related to chronic illnesses, such as HIV, interferes with disclosure, treatment and positive health-related behaviours, adding to the burden of illness, then one must begin to investigate its “affective climate”, to return to Race’s contention.

In this article, I am interested in following Race’s insights into the composition of a common negative affective climate surrounding HIV prevention and care – “fear, shame, secrecy, suspicion, rejection and avoidance” – while also exploring Hutchinson and Dhairyawan’s recent claim that *shame*, in particular, is central to HIV-related stigma. Hutchinson and Dhairyawan argue that to investigate HIV-related stigma without also investigating shame, “is to miss much of importance; it is akin to studying ‘threats’ as social phenomenon, while ignoring the fear that people have in response to those threats” (Hutchinson & Dhairyawan 2017b, 225). Of course, the association between shame and stigma is nothing new, and Hutchinson and Dhairyawan are certainly not the first thinkers to posit connections between HIV-related stigma and shame (Bennett et al. 2016; Halperin & Traub 2009; Skelton et al. 2020). It is often taken for granted that stigma causes experiences of negative self-conscious emotions, such as shame, embarrassment, humiliation and disgrace. In fact, some theorists posit shame to be the result of “self-stigmatisation”, or the “internalisation of stigma” (Skelton et al. 2020, 2). However, many theorists and researchers use the term “shame” to signal the on-going experience of stigma, but do so without explicitly exploring or theorizing the relationship between stigma and shame. These terms are often used interchangeably, while implicitly taking for granted a connection between the *emotion* and *experience* of shame and the *social attribute* of stigma.

My aim in this article is to give a clear account of how the experience of shame, particularly shame anxiety, is central to understanding how stigma causes harm, especially in experiences of chronic illness such as HIV. As such, I follow Hutchinson and Dhairyawan’s intuition that *shame* is not only part of the affective climate when considering HIV disclosure, prevention and care, but also one of the core mecha-nisms within HIV-related stigma that leads to distress, harm and an increased burden of illness. In this article, I will focus on accounts and testimony from gay men who are living with HIV. In doing so, I by no means intend to further entrench the debunked association between HIV and gay male populations. I draw on the case of Gareth Thomas’s HIV disclosure largely because of its contemporary and high-profile status within a UK context. In addition, it should be noted that HIV stigma for gay men is compounded by the stigma and shame historically associated with homosexuality and the “homosexual lifestyle” (Race 2021). My analysis of shame anxiety, as it is related to HIV disclosure, in particular, and stigmatised invisible chronic illness more generally, is relevant to other populations and groups living with HIV, including those with other intersecting stigmas, such as sexuality, gender, race, poverty and addiction.

I will begin by discussing the relationship between stigma and shame, engaging with Eve Kosofsky Sedgwick’s account of shame (Sedgwick 2003) to demonstrate the inherent relation between social bonds and shame experiences. I will then draw from the recent high-profile case of Welsh rugby player Gareth Thomas’s disclosure of his HIV status.^2^ I will draw on the moving and powerful first-person narrative shared by Thomas with the public through a video released via his Twitter profile, along with subsequent media interviews.^3^ Thomas’s narrative not only offers a visceral and honest account of the *lived experience* of stigma, but also reveals the common affective climate, identified above by Race, associated with HIV. I will argue that his account of living with stigma demonstrates stigma’s structural relation to shame. I will offer an account of “shame anxiety”, borrowing and extending the term from Stephen Pattison’s discussion of chronic shame (Pattison 2000, 85). I will then draw from resources from Edmund Husserl’s classical phenomenology (Husserl 1977, 2001) and Sara Ahmed’s work in *Queer Phenomenology* (Ahmed 2006), in order to investigate how shame anxiety manifests in bodily lived experience through the structure of the “horizon”, exploring the possibilities, or non-possibilities, of queer shame in such an experience. I will finish with reflections on how shame anxiety can act as a barrier to the effective delivery of health services for those with stigmatised chronic illnesses and, furthermore, why the experience of shame anxiety might be useful to consider when delivering health services.

## Stigma and shame

We generally use the term “stigma” to describe “the degrading marks that are affixed to particular bodies, people, conditions and places within humiliating social interactions” (Tyler 2020, 8). Inflicting a stigma is convenient as it immediately marks an individual, or group of individuals, out as “abnormal”, “unacceptable” or “inferior” and indicates that it is permissible to treat them as less than fully human. Stigmas are inflicted silently and invisibly through the social norms and political machinations of a dominant social group that mark out certain behaviours, physical characteristics or circumstances as unacceptable or deviant and, therefore, inferior. Stigma works both to isolate the one who is marked out as “inferior” and to build a sense of unity and commonality among those who remain in the fold.

Erving Goffman’s classical 1963 study *Stigma: Notes on the Management of Spoiled Identity* gives an account of stigma that precisely highlights stigma’s relationship to physical and behavioural characteristics within interactional social relations where society is organised along the lines of inclusion and exclusion: the “normal” and those with so-called “spoiled identities” (Goffman 1990). Goffman defines stigma as an attribute which an individual holds that is “deeply discrediting”; it is something which disqualifies a person from full social acceptance, marking them as defiled, deviant or non-normative by the “normals”, i.e., the dominant social group (Goffman 1990, 13, 15). Individuals bearing a stigma understand themselves to be inferior and discredited within the dominant social order, even if they themselves do not share this belief regarding their inferiority. Goffman identifies various types of stigma, many of which are health-related (“abominations of the body” as he calls them (Goffman 1990, 14)). Following Goffman’s insights, stigma has become a useful concept in health research.^4^ It is a means to understand the social impact of illness (Weiss, Ramakrishna & Somma 2006), or, in other words, how the experience of an illness may be concomitant with a range of social phenomena, such as discrimination, judgement, social exclusion, vilification, ostracism, labelling, status-loss, prejudice, and unfair treatment, among others (Link & Phelan 2001, 377).^5^

HIV has been subject to “exceptionally high levels” of stigma (Parker & Aggleton 2003, 13), where long-standing associations between homosexuality and social depravity have rendered the experience of contracting HIV profoundly stigmatizing. While HIV can now be successfully managed as a chronic condition, and societal attitudes have changed significantly, it still “remains one of the most stigmatised health conditions to date” (Skelton et al. 2020, 2). Often, those affected by stigma are deemed to be undeserving, perhaps unworthy of medical care; they are relegated to the margins of society for fear of contagion, both physical and moral. Ample evidence demonstrates that stigma can significantly worsen the burden of illness, impacting on health seeking behaviours, causing personal and social harm, and exacerbating existing social and health inequalities. In the case of HIV, for instance, the physical or functional limitations of illness are not what an individual deals with on a daily basis. Instead, what they are confronted with on a daily basis is societal and social responses to the illness and its associations (Susman 1994). By investigating stigma we can thus determine how and where power and domination are circulating within the realms of health, illness, medicine and healthcare (Parker & Aggleton 2003), and the effect of these power relations on individuals.

A health-related stigma may be visible, or “evident on the spot”, such as a disability or disfigurement, marking an individual immediately as “discredited” in the social order (Goffman 1990, 14). It may, however,also be not “immediately perceivable”. In other words, some health-related stigmas may be easily concealed or hidden, such as is often the case with HIV. In the latter case, the individual bearing the stigma is not yet discredited, but “discreditable” (Goffman 1990, 14), suspended with the interminable anticipation of judgment and social exclusion should their condition be discovered or revealed.^6^ Goffman quotes at length from Sullivan to illustrate aspects of the emotional texture of being *discreditable*:

The awareness of inferiority means that one is unable to keep out of consciousness the formulation of some chronic feeling of the worst sort of insecurity, and this means that one suffers anxiety and perhaps even something worse … The fear that others can disrespect a person because of something he shows means that he is always insecure in his contact with other people; and this insecurity arises … from something he knows he cannot fix. (Goffman 1990, 24)

An enduring awareness of one’s inferiority in the dominant social order manifests as “chronic” feelings of “fear” and “anxiety” in one’s contact with others, stemming from the risk of being judged, rejected and socially excluded. The affective register of being discreditable, I contend – in part following Hutchinson and Dhairyawan – is thus that of anticipated *shame*.

The association between shame and the *experienced* aspects of stigma is posited in some health literature. However, what is surprising is that in much of the current literature regarding health-related stigma, shame does not even get mentioned (Weiss, Ramakrishna & Somma 2006; Parker & Aggleton 2003). When shame is discussed, there is still a lack of consensus regarding whether shame is a component of stigma, or a related, but separate experience (Nyblade 2006, 338; Bennett et al. 2016).

In contrast to a broad lack of clarity regarding the relationship between shame and stigma, Graham Scambler explicitly discusses shame as a key experiential component to stigma in his article “Re-Framing Stigma”. Scambler aims to describe how living with a stigmatised illness impacts on the “biographies of those diagnosed” (Scambler 2004, 33), shifting the analysis to a first-person perspective. Scambler discusses “felt stigma” as a means to differentiate the experiential aspects of stigma from the social phenomena attributed to it. He suggests “felt stigma” has two referents: first, “the *shame* associated with” being reduced to the condition (e.g., “being epileptic”), and second, “the *fear of encountering enacted stigma”*, where “enacted stigma” refers to acts of discrimination on the basis that someone is perceived to be socially and culturally unacceptable (Scambler 2004, 33). Enacted stigma is, for Scambler, synonymous with “shaming” (Scambler 2018, 53). Scambler’s positing of a necessary relation between shame and the experience of stigma resonates with insights of other thinkers (Bennett et al. 2016; Hutchinson & Dhairyawan 2017a) who posit that stigma and shame are internally related, where shame is one of the possible, and perhaps necessary, experiential consequences of the social phenomena associated with being stigmatised (e.g., discrimination, prejudice, unfair treatment, ostracism, labelling, etc.).

Goffman himself points out that “shame” is a “central possibility” in experiences of stigma (Goffman 1990, 18). However, Goffman’s understanding of shame, as it is briefly elucidated, characterises shame as a self-evaluative response, “arising from the individual’s perception of one of his [sic] own attributes as being a defiled thing to possess” (Goffman 1990, 18), wherein an individual has internalised societal norms and comes to perceive themself *as stigmatised*, or as being defiled or lacking. This conception of shame corresponds to a common psychological formulation of shame as arising when there is a perception of a discrepancy between the ideal self and the actual self (Scheff 2000, 97). This definition of shame is highly individualizing, relegating shame to the private psychological sphere.

Shame, of course, is never an entirely private affair. Any experience of shame is personal and embodied, while also necessarily social and political: embedded in relational dynamics with others, while shaped by the norms and mores of one’s cultural and political milieu (Dolezal 2015a). To understand why and how shame has the potential to be part of all experiences of stigma, particularly bodily stigma, a more expansive understanding of shame is required: one that positions shame in human experience as centrally concerned with our inherent bodily vulnerability, sociality and need for belonging, or, in other words, as centrally concerned with our *meaningful connections to others.*

Eve Kosofsky Sedgwick offers such an account of shame, drawing from child development literature to demonstrate that shame arises when the “interpersonal bridge” is threatened (Sedgwick 2003, 36). Shame, as Sedgwick draws out in relation to Silvan Tompkins’s affect theory, is an embodied response which signals to the subject that their connections to others are under threat, or that social bonds may be severed.^7^ As Sedgwick notes, “shame both derives from and aims towards sociability” (Sedgwick 2003, 37). Understanding shame in this manner, means that while not all shame is related to stigma, all stigma is infused with the potential for shame. Stigma is precisely about one’s identity and position in social relations: the fear of losing social bonds and being positioned as one of “them” in relation to “us” through discriminatory practices. As a result, being on the receiving end of stigma, or being stigmatised, necessarily invokes the structural experience of shame as outlined by Sedgwick: one becomes vulnerable through the threat to the social bonds one has with those who can ensure one’s social, and perhaps physical, survival.

While a person may be stigmatised for a discrete part of their identity (e.g., an illness, the colour of their skin, their sexuality), it is important to note that stigma is never contained to the attribute in question, but instead attaches itself to the whole person. As a result, stigma envelops and affects the global self. As Goldin notes in relation to HIV, “stigma has tended to define the bearer rather than the sign carried by the bearer, and the bearer becomes known by the disease itself” (Goldin 1994, 1360). The bearer’s whole identity becomes defiled, discredited, *shameful.* In parallel, the shame associated with stigma is not limited to the stigmatised attribute, but comes to affect an entire identity. As Sedgwick notes: “The forms taken by shame are not distinct ‘toxic’ parts of a group or individual identity that can be excised; they are instead integral to and residual in the processes by which identity itself is formed” (Sedgwick 2003, 63). Having a sense of self that is “tuned most durably to the note of shame” (Sedgwick 2003, 63) as a result of the stigma associated with one’s illness or disease, has concrete consequences for how one not only experiences one’s illness, but also how one constructs one’s self image and identity, and conducts oneself in relation to others.

In this regard, Sedgwick is optimistic about shame. She posits shame as the primary queer affect and celebrates its positive potential for motivating queer creativity. She argues that shame can generate a positive space for identity, signalling a collective departure from the sphere of “ordinary” power relations which mark queer identities as deviant (Sedgwick 2003).^8^ However, I believe the positive potential for queer shame is complicated by the manner through which shame actually manifests (or more accurately, does not manifest) in lived experience as a result of stigma. My contention is that if we want to understand how health-related stigma, and its related social phenomena, are experienced by an individual and how this might impact on health-related behaviour and health outcomes, then we must understand how stigma-related shame is often *experienced as non-experienced*, or, as we shall see, as a “present absence”.

## Shame anxiety in HIV disclosure

On September 14, 2019, Welsh rugby player Gareth Thomas uploaded a powerful and emotional video onto Twitter where he publicly disclosed his status as HIV-positive. Thomas came out as gay in 2009 and is believed to be the first British sportsperson to go public about living as HIV-positive. Gareth Thomas’s testimony is a first-person account of the experience of living with a highly stigmatised invisible chronic illness, and the affective toll of concealing the illness. Thomas reveals that he was forced to publicly confess his HIV status as a result of a journalist having discovered his health status and revealing his secret to his parents (Godfrey 2020). While Thomas was able to secure an injunction to prevent the publication of a news story about him being HIV-positive, he decided to make a public statement in order to dispel the possibility that his HIV status would be revealed without his consent. In his emotive testimony in the Twitter video and subsequent media interviews and a BBC documentary, Thomas describes what it is like to live with the fear of being found out, evoking the twofold structure of Scambler’s “felt stigma” (shame and the fear of shameful discrimination), while also highlighting Sedgwick’s account of shame as centrally concerned with our meaningful connections to others. Thomas’s account powerfully describes his reasons for concealing his illness, and the anticipated consequences of its revelation. I draw on Thomas as a case study, in the phenomenological sense, to highlight the experiential structure of what I will discuss as “shame anxiety”. While I recognise that Thomas’s account is not representative of all individuals, or even of gay men, living with HIV,^9^ I believe his account provides useful insights into a common “affective climate”, identified by Race, central to the experience HIV diagnosis, disclosure and treatment. Hence, my interest in his account concerns the experience of living with an invisible chronic illness, or in other words, Thomas’s account of his experience of his stigmatised condition *before* publicly revealing his secret. I quote here from Thomas’s own account of his experience, bringing together quotations from several sources:

“I want to share my secret with you … I’m living with HIV. Now you have that information that makes me extremely vulnerable but it does not make me weak.”^10^

“I’ve felt shame and keeping such a big secret has taken its toll.”

“I had a fear people would judge me and treat me like a leper because of a lack of knowledge. I was in a dark place, feeling suicidal. I thought about driving off a cliff.”

“To me, wanting to die was just a natural thought and felt like the easier way out, but you have to confront things.”

“But the truth is I’m still scared even now of people finding out I’m living with HIV and I’m s****ing myself and feel petrified about what the reaction will be, because we still live in an era where HIV is not spoken about.”

“I thought that if people knew about me being HIV positive they wouldn’t want to breathe the same air as me, they wouldn’t want to drink from the same cup as me and if I walked into a coffee shop everyone would just walk out because they’d be so scared of being infected by me.”^11^

“When you have a secret that other people know about it makes you really vulnerable towards them. And I just felt like I had no control over my own life.”

“It has all [receiving treatment] been shrouded in a sense of shame and from me entering the clinic to leaving always feels like a blur.”^12^

“I chose to fight and break the stigma around this subject.”^13^

“The reason I’m doing this is because I want to remember what it’s like to live again; I want to remember what it’s like to feel free.”^14^

The recurrent themes in Thomas’s testimony regarding the disclosure of his HIV status and his on-going treatment are fear, vulnerability, shame and stigma. Thomas’s fear of his secret being found out, is given its affective force by his anticipation of social vulnerability and shame, of being judged and possibly ostracised by others. Race identifies a similar constellation of concerns regarding HIV disclosure, where HIV-positive men are reluctant to reveal their health status (in this context to potential sexual partners) because of fears of “rejection and humiliation”, along with fear “that others might treat them contemptuously, judge or demonise them for being HIV-positive” (Race 2018, 64). These “affective complications” (Race 2018, 62) add to the overall negative “climate”of “fear, shame, secrecy, suspicion, rejection and avoidance” (Race 2018, 117) that clearly also characterise Thomas’s vivid and affective descriptions of his experiences.

In Thomas’s account, the compulsion to contemplate suicide is not linked to his illness (which he initially assumed was potentially terminal), but instead to his fear of social judgement and social exclusion. Or, if we follow Sedgwick’s account of shame as being inherently linked to vulnerability and sociality, his fear of shame. This association between fearing shame and suicide is by no means arbitrary, nor extreme; there are very high stakes involved when one feels socially vulnerable and has a sense that one’s social bonds are irreparably compromised. As Jane Megan Northrop notes, in cases of stigma “social death and actual death are imminently convergent” (Northrop 2012, 105). Tomkins concurs, noting that “loss of face [can] be more intolerable than loss of life” (Tompkins 1995, 136). Research confirms these insights, where shame, and unequal distributions of shame, can be strongly related to self-harm and suicide. When “shame becomes visible – when it is acknowledged – it can be felt more intensely” by those from stigmatised communities (Chandler 2020, 42). As a result, avoiding potential instances of shame, through concealing one’s stigmatised condition, can feel like a life-saving measure. When this concealment is no longer possible, suicide may seem like a logical option.

Thomas’s account further highlights the structures of shame and shameful anticipation inherent to felt stigma. Thomas describes explicitly the *actual* shame he feels as a result of his condition and his on-going treatment, while also describing the *potential* shame he fears as a result of being found out, of his secret being revealed (by the journalist) and the subsequent judgement and discrimination that would ensue. It is living with this ongoing “fear” that led Thomas to feel that he had no “control of [his] own life” and as though he didn’t “feel free.”

The consequences of living with the anticipation of shameful exposure in relation to the revelation of HIV status are eloquently described by Kane Race in a semi-autobiographical essay about his experience of receiving an HIV diagnosis in 1996, the same year that combination anti-retroviral therapy transformed HIV from a death sentence to a chronic and manageable illness (Race 2021). In Race’s essay, we see striking structural similarities to Thomas’s account:

Soon after receiving my HIV diagnosis I made an appointment with a general practitioner with years of specialist experience in HIV care … “Don’t tell your parents,” was the first piece of advice he offered me … I did hold off on telling my parents for some time … But living in constant fear of imminent exposure with a stigmatized, sexually humiliating condition takes its toll … It was only a matter of time before they discovered my dirty secret, I figured. In the narrative that dominated my parental imaginary, HIV operated as vindicating proof of sexual depravity: conclusive evidence of the wrongness of homosexuality and the foolish lifestyle I was leading. I imagined my parent’s anger, disappointment, and deep shame on discovering that their only son had succumbed to the logical outcome of the homosexual lifestyle they had warned me against repeatedly. I had wasted my life, wasted everything, betrayed every hope they had invested in me. This apprehension of a wasted life filled me with a sense of impending catastrophe …. Intensely conscious of the shameful disappointment I had become, I lived in constant fear of public humiliation and unwanted exposure … [as a result of the] intense sexual shame that the possibility of having my HIV status outed to my parents unwittingly provoked in me (Race 2021).

For Race, the “constant fear” of exposure and sense of “impending catastrophe” was intimately linked to a negative evaluation of the self through the eyes of an “other”, in this case an “other” dominated by his “parental imaginary”. Race describes how shame is anticipated: he may at any moment suffer humiliating exposure and be judged for his “sexual depravity” and subsequently rejected. The explicit anticipation of shame, related both to his sexuality and HIV status, comes to be a defining feature of his lived experience. Likewise, in Thomas’s account, we see a fear of exposure and judgement leading to social exclusion: “I had a fear people would judge me and treat me like a leper”; “I’m still scared even now of people finding out … feel petrified about what the reaction will be.” Both Race and Thomas, I argue, give accounts of the structure of stigma-related “shame anxiety”, where shame anxiety is understood, following Pattison, as an “anticipatory anxiety about the imminent threat of being exposed, humiliated, belittled or rejected” (Pattison 2000, 85).

Living with shame anxiety does not mean that shame is experienced continuously, but instead that the threat of shame is predominant and persistent. There is a crucial distinction between the *actuality of shame* and the *potentiality of shame.* Living with a stigmatised invisible chronic illness involves both actual shame and potential shame, as Thomas’s testimony reveals. Following Scambler’s structure of felt stigma, there is the shame related to one’s illness (actual shame) and the fear of enacted stigma where one is afraid of social rejection through discriminatory actions (potential shame). Shame anxiety is the experience of *potential shame* or the shame that is anticipated when one is afraid that one’s concealed illness will be discovered and that one will be judged, scorned or ostracised. What is interesting in the experience of shame anxiety is that shame itself may not be present in the affective landscape. Instead, it manifests precisely as a “fear” of exposure, or a “chronic feeling of the worst sort of insecurity,” to invoke Sullivan on stigma again.

As a result, shame anxiety is frequently characterised by the *absence*, rather than the *presence*, of shame. Instead of the searingly painful selfconsciousness that accompanies episodes of actual shame, the experience of shame anxiety can render shame invisible, both to the self who is experiencing it, and to others around them. Shame is fastidiously avoided through control of one’s behaviour, interactions and actions, or through the avoidance of certain encounters, situations or conversations. As Pattison notes about individuals who live with shame anxiety: “They live their lives trying to avoid occasions and relationships that might provoke painful shame experiences” (Pattison 2000, 83). In the cases of Thomas and Race, shame is avoided through secrecy and concealment in certain situations and with certain individuals. Understood in this way, shame anxiety can be characterised as a persistent *absence of shame*, which is nonetheless experienced as *present* through anticipation. And this “present absence” has tangible consequences in terms of how one experiences one’s behaviour, actions, social relations and broader world.

## A phenomenology of shame anxiety

Understanding shame anxiety as a “present absence” gives us inroads to describing the experience through a phenomenological register and therefore to understanding how it manifests in lived experience. In Edmund Husserl’s classical phenomenological account of consciousness, he demonstrates that all human experience is characterised by experiences of “present absences”: what is “strictly non-experienced but necessarily also meant” (Husserl 1977, 23). Husserl uses the concept of the “horizon” to capture this idea, borrowing meaning from how we understand horizon in the usual sense as the limit of what is visible or perceptible (Geniusas 2012, 1). The horizon is not a fixed location, but a shifting limit of possible experience. As such, the horizon acts experientially as a real limit point demarcating what is experienced from what is “non-experienced”, or a “non-presence” but nonetheless within the realm of possibility and, hence, in some sense part of experience.

Hence, “horizons”, for Husserl, “are ‘predelineated’ potentialities” in our experience (Husserl 1977, 45). Due to its temporality, our experience has a horizonal structure. In an act of perception for example, when looking at a table, I will at any given moment only be able to see one side of the table. If I am looking down at the table, the underside of the table, for instance, is part of the table’s horizonal possibility. In other words, in an act of visual perception, the non-visible aspects of the table remain “co-present” but in some sense absent. Hence, perception involves a constant relationship between presence and absence, or “fullness and emptiness” to use one of Husserl’s formulations (Husserl 2001, 44). As Husserl sees it, all conscious experience is characterised in this horizonal way, as a blend of absence and presence, or fullness and emptiness. Any experience is surrounded by horizons of possibility – spatial, temporal, experiential – that are co-present, but currently absent.

In her book *Queer Phenomenology*, Sara Ahmed takes up Husserl’s notion of the “horizon”. She discusses how the horizon is more than what is currently absent (but possible) in experience, and instead argues that the horizon “is what gives objects their contours” and “makes them available … for me” (Ahmed 2006, 55). Extending the concept of “horizon” both existentially and politically, Ahmed argues:

what ‘comes into’ view, or what is within our horizon, is not a matter simply of what we find here or there … What is reachable is determined precisely by orientations that we have already taken. Some objects don’t even become objects of perception, as the body does not move toward them: they are ‘beyond the horizon’ (Ahmed 2006, 55).

What this means is that horizons are not neutral possibilities in an objective world, but instead are shaped by our situations, orientations, histories and emotions. A table becomes an object of significance for me only if I have a history of relating to tables or of activities that may require them. Ahmed quotes Paul Schilder: “The emotional configuration determines the distance of objects from the body” (Ahmed 2006, 54–55). In other words, our situations, histories and their concomitant affects, make certain possibilities salient in our experience while invisibilising others.

When thinking about shame anxiety as a present absence, we see precisely the horizonal structure outlined by Husserl and nuanced by Ahmed. Of course, shame is not a spatial object (like a table), but instead an affective experience. Nonetheless, the horizonal structure is parallel. In shame anxiety, shame is absent, however it is also “present” as a “predelineated” possibility. This occurs through a process that can be understood through Ahmed’s conception of “orientations”. We become “oriented” to shame, and familiar with its contours, through understanding and experiencing social norms. If our identity, appearance, or health status continually violates dominant social norms, then we may become “oriented” to the anticipation of shame. A fear of shameful exposure may come to chronically dominate our experience.

To deploy Husserl’s language of the horizon: in experiences of shame anxiety, shame is “non-experienced” but “necessarily also meant”. As both Thomas’s and Race’s accounts demonstrate, the fear of shameful exposure (of a shame which is presently absent and which may remain indefinitely absent), comes to structure experience: their expectations, feelings, thoughts, actions and social relations are impacted by the shame experience which exists “just out of awareness” but nonetheless feels like a real and imminent possibility.^15^ Of course, certain contexts, situations and communities open up, or close down, horizons of possibilities in different ways. While shame anxiety may come to be part of the fabric of lived experience, it may also be heightened in particular spaces or contexts where one feels an intensified sense of self-consciousness, difference or fear of exposure. In these spaces or contexts, one’s horizons of possibility may shrink to circle tightly around the fear of shameful exposure. In other spaces or contexts, where one is among “kin” – others who share or understand one’s identity, circumstances or health-status and has a sense of acceptance and belonging, one’s horizons of possibility may feel more capacious. In these instances, shame anxiety may be attenuated.^16^

## Shame anxiety and health outcomes

Living with the permanent anticipation of shameful exposure is clearly comprising for an individual. This persistent “orientation” towards shame, as an object “of thought, feeling and judgement” has consequences (Ahmed 2006, 56). As Pattison notes, those who are “shame-prone, shame-vulnerable or chronically shamed … often perceive themselves to be weak, inferior, ineffective, defiled, defective, unlovable, diminished, depleted failures” (Pattison 2000, 110). Living with such negative self-conceptions has concrete consequences for lived experience. As Philip Wüschner notes, the experience of consistent shame “reduces the *hodological space* to a suffocating level, minimising it to the very ground a person stands on” (Wüschner 2017, 103). In other words, the physical, social and affective spaces which contain the possibilities for action, engagement, and sociality are reduced so drastically – to use Wüschner’s metaphor to a point nearing suffocation – that the individual can feel immobilised. In phenomenological terms, the horizons of possibility with respect to one’s actions, sociality and life chances, may become drastically reduced as one continuously implements strategies to circumvent shameful exposure and this is compounded by intense negative feelings regarding oneself and one’s social standing. Thomas’s testimony offers a powerful account of precisely this constriction; his motivation for confessing his HIV status is to lift the burden of shame, and in doing so, he invokes a resumption of life and freedom: “I want to remember what it’s like to live again; I want to remember what it’s like to feel free.”

Not only may chronic shame anxiety affect one’s behaviours, actions and interactions with others, it also has implications for one’s sociopolitical position. As Jill Locke notes, “repetitive shame wears on the subject’s ability to inhabit, much less participate in and transform, the political world. Weariness readily translates into civic invisibility – a withdrawal from public life for some, a failure to engage in the first place for others” (Locke 2007, 153). In this way, chronically anticipated shame can be disempowering both socially and politically, which has obvious significance when considering highly stigmatised and politicised conditions such as HIV.

It is precisely these personally and politically disempowering effects of shame that are being challenged in the celebration of “queer shame”,^17^ where queer scholars, following Sedgwick, argue that “instead of denying or repressing queer shame [we] should aim to accept our differences from the dominant majority, acknowledge our shame, and make creative use of that shame” (Morrison 2015, 18). This “reframing” of shame is intended as an identity affirming move, which carves out a space of acceptance and non-normativity. Instead of being “stigmaphobes”, trying to extract queer contaminants and to blend into conventional and normative life, those who can claim their shame aim to live as “stigmaphiles” (Warner 1999, 43), “accepting shame and stigma as motivating forces in their lives” (Morrison 2015, 22). While this aspiration towards queer defiance in the face of shame and stigma is compelling, it is worth thinking through what the genuine possibilities of reframing queer shame are for those living with chronic shame anxiety.

While some may have the inner resources to reframe shame and stigma productively, many who are affected by the politics of shaming and the damaging effects of stigma, may not have the social capital nor the material or psychological resources to effectively name their shame in order to resist it. As Lisa Guenther notes, “shame attacks the very resources that one would need in order to resist shame, and to put in question the mechanisms that produce it and distribute it unevenly among subjects” (Guenther 2011, 24). Recalling the phenomenology of shame anxiety, shame is often hidden in one’s experience, manifesting only as an anticipation of shame: a fear or anxiety about the potential loss of social bonds and social standing. The chronic anticipation of shame may itself erode the potential for a positive reframing of shame, as shame continuously eludes our present experience, leaving us to register instead an undermining sense of fear, anxiety and personal inadequacy, along with a strong sense of wanting to hide or withdraw. In addition, not all individuals will be able to take the social and political risks associated with queering their shame. When we consider the material and social conditions of many individuals and groups living with HIV – for instance people living with other axes of disempowerment or marginalisation (e.g. race, gender, class, poverty, addiction) or who are simply burdened with the stress of ill-health or routine discrimination – there are perhaps few who feel they can take these additional risks.

Of course, this is not to suggest that resistance, activism and solidarity is impossible for those who experience chronic shame anxiety. As Stangl et al. note, when considering stigma, “the interconnections between power and vulnerability … are fluid and complex” (Stangl et al. 2019, 4). There always exist concrete horizons of aspiration: possibilities for resistance, re-framing, reclaiming power, and fighting social inequalities and unfair treatment. Jill Locke posits the idea of “unashamed citizens” as those who expose the “invisibilities, dominations, and inequalities” that are naturalised in social relations. These unashamed citizens, Locke argues, call out the shame that is (wrongly) imposed on them and, in doing so, act as “radically democratic” agents who “resist and disavow the terms of shame” to agitate for more inclusion, compassion, and justice (Locke 2016, 173).^18^ These horizons of aspiration are important, and some of us can embrace the positive potential of queer shame, along with other forms of non-queer shame resistance, for creativity and identity formation. Nonetheless, it should be remembered that within these personal and collective projects of transformation, the negative effects of shame in broader socio-political contexts may not be eliminated, or even attenuated, for all those who are stigmatised, marginalised or (a)shamed. In short, not being able, or willing, to embrace queer shame should not itself become another source of shame/shaming.

In the context of considering HIV and its related stigma, it is important to theorise how shame (actual and anticipated) can impact on health outcomes in various manners. There is an emerging literature exploring the link between the experience of shame and health outcomes, positing shame as an “affective determinant of health” (Dolezal & Lyons 2017). The suggestion is that shame, as a chronic stressor, “may orchestrate specific patterns of psychobiological changes” in the body which can exacerbate physical conditions (Dickerson, Gruenewald & Kemeny 2004, 1191). For example, in a study of HIV-positive patients, experiences of shame and threats to one’s social bonds correlated with illness progression and mortality. Interestingly, researchers found that experiences of shame can “predict disease-relevant immunological and health outcomes in HIV” (Dickerson, Gruenewald & Kemeny 2004, 1191).

In addition to possibly contributing to the burden of illness, the experience of shame anxiety also significantly impacts on health-related behaviours. There is evidence that the anticipation of shameful exposure in healthcare contexts can lead to a range of behaviours and outcomes which can negatively affect one’s health status and treatment (Dolezal & Lyons 2017, 259, Dolezal 2015b). Hutchinson and Dhairyawan highlight five ways that shame negatively impacts the treatment of HIV:

1. Shame can prevent an individual from disclosing all the relevant facts about their sexual history to the clinician.
2. Shame can be a motivational factor in people living with HIV not engaging with or being retained in care.
3. Shame can prevent individuals from presenting at clinics for STI and HIV testing.
4. Shame can prevent an individual from disclosing their HIV (or STI) status to new sexual partners.
5. Shame can serve to psychologically imprison people, it makes the task of living with HIV a far more negative experience than it should, or needs to, be (Hutchinson & Dhairyawan 2017a, 1).

While Hutchinson and Dhairyawan’s analysis of shame’s impacts on people living with HIV is insightful and important, what their theoretical study misses is the way shame related to health stigma is *experienced.* As we have seen, a phenomenological analysis reveals that shame is often experienced in the form of a present absence. It is often the *potentiality of shame* in the form of a *fear of shameful exposure*, rather than *actuality of shame*, that is a strong force to consider in health-related encounters, especially for highly stigmatised invisible chronic illnesses such as HIV. This distinction between potentiality and actuality is significant because the *present absence* of shame may be difficult to capture empirically. As Susie Scott notes, experiences which are “unmarked” or characterised by “non-presence” are often sociologically neglected (Scott 2018). This may be the reason why experiences of shame anxiety can remain undetected, invisible or difficult to articulate in socio-political and empirical analyses of health-related stigma.

## Conclusion

While health professionals are aware of how stigma can have negative effects in terms of how one experiences one’s illness, they can have difficulty directly addressing the effects of stigma in their clinical encounters. By contrast, if health professionals recognise shame and shame anxiety as structurally related to stigma, then this may lead to concrete strategies and interventions that address some of stigma’s negative effects on health outcomes. Indeed, Bennett et al. suggest that “shame may … be more readily addressed than stigma by clinicians” (Bennett et al. 2016, 88). This, of course, may be for the simple reason that clinicians are more readily able to address, and work through, an individual’s experience of shame, whereas addressing and resolving stigma would entail structural, social and political change well beyond the scope of individual clinical encounters.^19^ A phenomenological analysis of the shame related to stigma, manifesting as shame anxiety, allows us to consider shame as a “present absence”, and to theorise how anticipated shame can be a powerful force in health contexts and with respect to health-related behaviours. Shame may not register as part of experience, either by patients or by healthcare providers, but may nonetheless be a key part of the horizon of experience, shaping an encounter or interaction as a result of shame avoidance strategies due to the ongoing fear of shameful exposure, as Thomas’s and Race’s powerful accounts of keeping the secret of their HIV status demonstrate. Contradictorily, it is the *absence* of shame that becomes significant in experience. Theorizing the potential for shame, or shame anxiety, as a structural possibility in experiences of stigma may be a powerful means to understand the mechanisms through which stigma can cause distress and exacerbate negative health outcomes.

## Notes

1 In my use of the terms “phenomenology” and “phenomenological” I refer to the philosophical tradition of phenomenology, which originated with Edmund Husserl’s declaration of phenomenology as a philosophical method for describing the structures of first-person lived experience (Husserl 2002) generalisable or universalizable across all human subjects. I offer this clarification as the terms have slightly different significations across different disciplines: in the social sciences a “phenomenological analysis” can refer to an individual subjective account from the first-person perspective without any concern for structures of experience that can be universalised. My aim is to provide a phenomenological account of shame anxiety, which is generalisable across a variety of subjects, as a means of understanding how stigma registers in the textures of lived experience from a first-person perspective.

2 I am drawing from Gareth Thomas’s account in the tradition of the phenomenological case study (Zahavi 2010, see also Merleau-Ponty 2012, 112). I would like to acknowledge the influence of Bonnie Mann and her article “Shame, Femininity and Redemption” where she insightfully analyses the first-person testimony of Amanda Todd through a self-posted YouTube video in order to draw out some of the structural features of what she terms “unbounded shame” (Mann 2018).

3 Gareth Thomas’s video on Twitter “sharing his secret with you” - https://twitter.com/gareththomas14/status/1172979157762039810?s=20 (Accessed March 9, 2020) (Thomas 2019).

4 Goffman’s account of stigma has been hugely influential and offers useful insights into the relational and contingent aspects of stigma, primarily focussing on stigma’s personal and interpersonal manifestations. However, as Imogen Tyler argues, Goffman’s account of stigma is almost entirely politically vacuous; Goffman neglects to consider how stigma is connected to broader apparatuses of power and control and “excludes the fact that social relations are always already structured through histories of power (and resistance)” (Tyler 2020, 99). In short, Goffman’s account neglects the political dimensions of stigma, or, as Tyler argues in her reconceptualisation of stigma in *Stigma: The Machinery of Inequality*, how stigma is a political practice and an instrument of state coercion used to systematically oppress, marginalise and dehumanise certain individuals or groups. As Tyler points out, Goffman’s “interest is in *how* social rules work rather than in what they proscribe” (Tyler 2020, 99).

5 Stigma is by no means an easy phenomenon to define, especially when considering the many manifestations of health-related stigma. Going into the complexities of different types of stigma is beyond the scope of this paper, however Vickers offers a useful taxonomy of health-related stigma (Vickers 2000, 138–144). My contention regarding shame anxiety as the experiential component subtending stigma applies to the various categories of stigma outlined in Vickers’s taxonomy.

6 Jane Megan Northrop’s discussion of Hepatitis C gives an excellent example of an illness that can be concealed, but not entirely: one is legally obliged to reveal one’s illness e.g., when applying for jobs, visas, bank loans, or when presenting at a clinic or hospital for medical treatment. So while one’s illness can be concealed effectively, the anticipation of having to reveal it at some critical juncture may be ever present (Northrop 2017).

7 Drawing on Martha Nussbaum’s account of primitive shame (Nussbaum 2004) and Sartre’s account of pure shame (Sartre 2018), I argue elsewhere that shame is related to our inherent bodily vulnerability from early infancy (Dolezal 2017b), drawing conclusions similar to Sedgwick’s.

8 The possibilities for “gay shame” as an alternative to “gay pride” and as a positive affective space for gay and queer individuals are explored at length in David M. Halperin and Valerie Traub’s edited collection *Gay Shame* (Halperin & Traub 2009).

9 It has been pointed out that Thomas’s descriptions of his experience of being diagnosed with HIV are somewhat anachronistic, in that he, as one journalist puts it, describes “many of the fears of someone who came of age in the ‘80s and ‘90s” (Stroude 2020). As João Florêncio notes, “the late 20^th^ century was a time of fear,a time when the future was not only certain but it could likely also deliver us our worst nightmare in the shape of a lethal form of intimacy” (Florêncio 2020, 199). Thomas’s experience may not reflect the wider experience of some gay men in the UK and elsewhere who, for the most part, live with less stigma around sexuality and HIV status, and who may be more informed about how HIV can be managed as a chronic condition. Nonetheless, Thomas’s account resonates with affective accounts in more contemporary research findings among out and socially accepted gay men (Race 2018), while also demonstrating that, for many, perhaps in rural or conservative communities, the horizons of possibility are greatly constricted with respect to embracing a queer or gay lifestyle. In many places, and within many communities, homosexuality and being HIV-positive is still highly stigmatised and may result in high levels of discrimination and vilification, along with other stigma-related phenomena.

10 Thomas 2019.

11 The above quotes are quoted from Hill 2019.

12 Hill 2019.

13 Thomas 2019.

14 Davies 2019, from BBC video.

15 When considering the experience of gay men in relation to HIV, there is an interesting parallel to the “present absence” of shame related to HIV infection and disclosure, and what João Florêncio discusses as the “ghost of a future-presence that would always haunt our every sexual encounter” (Florêncio 2020, 198), or in other words, that gay men live with HIV either as an “actual infection” or a potential or anticipated infection.

16 I would like to acknowledge João Florêncio’s insightful comments on this article which helped me to formulate some of these ideas about differential horizons of possibility.

17 It should be stressed that I, by critically discussing “queer shame”, do not intend to suggest that shame related to HIV is queer, nor that HIV is an illness confined to gay or queer communities.

18 Elsewhere, I offer similar criticisms to Locke’s account, arguing that it is only those with the material and social resources that may be able to take the political and social risks inherent to becoming “unashamed” (see Dolezal 2017a).

19 Thank you to one of my anonymous reviewers for providing feedback on this point.
